# Exploring Factors Influencing Injury Severity of Vehicle At-Fault Accidents: A Comparative Analysis of Passenger and Freight Vehicles

**DOI:** 10.3390/ijerph17041146

**Published:** 2020-02-12

**Authors:** Jianyu Wang, Huapu Lu, Zhiyuan Sun, Tianshi Wang

**Affiliations:** 1 Institute of Transportation Engineering and Geomatics, Tsinghua University, Beijing 100084, China; jy-wang15@mails.tsinghua.edu.cn (J.W.); luhp@tsinghua.edu.cn (H.L.); wts18@mails.tsinghua.edu.cn (T.W.); 2 Beijing Key Laboratory of Traffic Engineering, Beijing University of Technology, Beijing 100124, China

**Keywords:** binary logistic regression, full or main responsibility, passenger and freight vehicle accidents, injury severity

## Abstract

The objective of this study is to find factors influencing the injury severity of vehicle at-fault accidents in Shenyang (China), and discuss the commonalities and differences between passenger and freight vehicle accidents. We analyzed 1647 traffic accidents from 2015 to 2017, in which motor vehicles were fully or mainly responsible, including 1164 traffic accidents caused by passenger vehicles and 483 traffic accidents caused by freight vehicles. Twenty influencing factors from the aspects of accident, driver, time, space and environmental attributes are analyzed to find their statistical connection with injury severity using the binary logistic regression model. For passenger vehicles, five influencing factors (side collision; illegal act while driving; hit-and-run; season and administrative division), showed statistically significant correlations with the injury severity. For freight vehicles, three influencing factors (illegal act while driving; season and administrative division), showed statistically significant correlations with the injury severity. Illegal act while driving is the only common influencing factor for the injury severity of both passenger and freight vehicle accidents. Side collision and hit-and-run are significant influencing factors for the injury severity of passenger vehicle accidents, but not for freight vehicle accidents. Season and administrative division present different results on influencing passenger and freight vehicle accidents. Based on these results, measures including driver education and road infrastructure improvement could be implemented to reduce the injury severity of accidents in passenger and freight vehicles.

## 1. Introduction

According to the World Health Organization, the number of road traffic deaths worldwide reached 1.35 million in 2016. In all age groups, road traffic injuries are the eighth most common cause of death, and the only non-disease lethal factor among the top ten causes of death [1]. In 2018, a total of 203,049 road traffic accidents occurred in China, which caused 63,772 deaths and 209,654 injured, with direct property damage of 121.313 million yuan. In order to reduce the number of deaths and property losses in road traffic accidents [2] it is therefore necessary to study the relevant factors affecting deaths during road traffic accidents.

The studies of motor vehicle accidents include accident frequency studies and injury severity studies [3]. In comparison with accident frequency, injury severity has gained more attention from scholars because it reveals the relationships between different influencing factors and deaths in traffic accidents. Motor vehicles can be divided into passenger vehicles and freight vehicles according to their different functions. They have different types of driving licenses, different transportation contents, and disparities in vehicle performance, which may cause differences on the factors influencing road traffic accident deaths. For cold cities, the road environment will show different states in different months, hence the environment could also have a great impact on injury severity [4].

Shenyang, with a population of 8.31 million and GDP of 629.4 billion yuan in 2018, is the capital city of Liaoning Province in China [5]. As Shenyang is in the northeastern part of China, there is roughly a 5-month wintertime period where roads are prone to unfavorable conditions such as freezing. Based on historical road traffic accident data in Shenyang, this study looks for the common and different factors affecting the deaths of passenger in traffic accidents and freight traffic accidents, and seeks to determine measures by which road traffic safety levels can be improved.

## 2. Literature Review

Factors influencing the injury severity of traffic accidents have been discussed by many scholars. Previous studies indicated that factors affecting the severity of road traffic accidents include the age of the motor vehicle driver [6], aggressive driving behavior [7], driving experience [8], drunk driving [9], weather [10], road infrastructure [11], hazardous materials transportation [12], accident circumstances [13], etc.

Passenger accidents and freight accidents show different rules due to the different operation contents. Previous scholars have built models for passenger and freight vehicle accidents. George et al. [14] investigated the severity of road traffic accidents for each model and established injury severity models for each type of vehicle. Kaplan et al. [15] studied the severity of bus accidents in the United States and he established a generalized, ordered logit model, determined the marginal impact of risk factors on the severity of bus accidents, and proposed vehicle standards and education. Kong et al. [16] explored the relationship between the collision speed of passenger vehicles and the risk of pedestrian injuries based on actual traffic accident cases in China. Islam et al. [17] analyzed the damage levels of single-vehicle (SV) and multi-vehicle (MV) large truck failures in rural and urban areas of Alabama. Considering the occurrence of vehicle accidents, four independent influencing factors of damage severity were studied by random parametric logit model. Torregroza-Vargas et al. [18] determined the variables related to the vehicle accidents involving truck drivers and established a logistic regression model. However, there is currently insufficient studies on the comparative analysis of passenger and freight vehicle accidents.

As for the research on cold areas. Pei et al. [4] analyzed the causes of traffic accidents, monthly distribution of traffic accidents, road types, and accidents in cold areas based on data collection, data processing, and field investigation. Wu et al. [19] gave the predicted values of the number of traffic conflicts and traffic accidents corresponding to different traffic volumes during cold and snowy periods in cold regions.

Scholars have adopted many methods and models to analyze the influencing factors of traffic injury severity, and the frequently used methods and models include Pearson chi-squared test method, hypothesis testing method, binary logistic regression model, multiple logistic regression model, ordered logistic model, CART, etc. Garrido et al. [20] used an ordered probit model to study the impact of multiple factors on the severity of motor vehicle traffic accident injuries. Theofilatos et al. [21] applied binary logistic regression model by setting the interpreted variable into two categories: death / severe injury (KSI) and minor injury (SI). Milton et al. [22] built a mixed logit model using highway damage data from Washington State. Vorkojovic et al. [23] performed a simple bivariate analysis using chi-squared, odds ratio, and 95% confidence interval to determine the risk of death, severe, and mild injury in three prognostic groups. Among all the methods and models, chi-squared test is usually used as a preliminary test of all influencing factors. Logistic regression analysis is usually used for deep analyzation of correlation and it contains binary logistic regression, ordered logistic regression and multiple logistic regression. According to the number of designed values of interpreted variable, a feasible logistic regression analysis model could be implemented. CART model is usually used for ranking the importance of different significant influencing factors.

## 3. Data and Methods

### 3.1. Data

According to the data of road traffic accidents from 2015 to 2017 provided by Traffic Management Department of Shenyang Public Security Bureau, 1647 traffic accidents in which motor vehicles were fully or mainly responsible are analyzed, including 1164 traffic accidents caused by passenger vehicles and 483 traffic accidents caused by freight vehicles. A set of influencing factors is established for the above accidents. Y is the interpreted variable indicating injury severity; X_i_ is the independent explanatory variable indicating influencing factors of injury severity. The influencing factors include five types of attributes, which are accident attributes, driver attributes, time attributes, space attributes and environmental attributes. The specific factors under each attribute are as follows:

(1) Accident attributes: type of motor vehicle (X_1_), frontal collision (X_2_), side collision (X_3_), rear-end collision (X_4_), accident liability (X_5_);
(2) Driver attributes: gender (X_6_), age (X_7_), driving years (X_8_), illegal act in driving (X_9_), owning a qualified driver’s license (X_10_), hit-and-run (X_11_);
(3) Time attributes: weekday or weekend (X_12_), season (X_13_), time interval (X_14_), rush hour (X_15_);
(4) Spatial attributes: administrative division (X_16_), location of road cross section (X_17_), physical isolation of road (X_18_), pavement condition (X_19_);
(5) Environmental attribute: weather (X_20_);

Five different types of attributes contain 20 factors that may significantly influence the severity of traffic accidents. Each influencing factor has different values, which represent different situations of accidents under this factor. Table 1 shows the definitions of different values of injury severity (Y) and influencing factors (X_i_). The percentage of each value is listed in Table 2. 

From Table 2, it is found that the percentage of non-death accidents are higher than that of death accidents for passenger vehicles. However, for freight vehicles, it is opposite. Additionally, for both passenger vehicle and freight vehicle accidents, man drivers count more than 90% of all drivers. 

### 3.2. Methods

In this study, since the interpreted variable (Y) has only two status (Y = 0, no death; Y = 1, death accident), binary logistic regression model is adopted to analyze the relationship between different influencing factors (Xi) and injury severity (Y). The mathematical expression of the probability of death accident occurrence is shown as the following equation:

(1) $$P(Y|X_{1},X_{2},\ldots .X_{16})=\frac{1}{1+e^{-\left(b+\sum_{i=1}^{16}a_{i}x_{i}\right)}}$$

where b is constant, and a_i_ is the coefficient of each explanatory variable. The probability equation can be transformed into the following form through logit transformation:

(2) $$\ln\left(\frac{P}{1-P}\right)=b+\overset{n}{\underset{i=1}{\sum }}a_{i}x_{i}$$

All the influencing factors of traffic injury severity are brought into the equation for calculation. The significance level of 0.05 is set as the indication of calculation and exp(B) means the ratio of possibility in comparison with the reference term causing the value of Y changes from 0 to 1.

## 4. Results

Based on binary logistic regression analysis, the results are shown in Table 3, five factors are significant for passenger vehicle accidents and three factors are significant in freight vehicle accidents. The following sections will describe the results in detail.

### 4.1. Passenger Vehicle Accidents

**Figure 1:**
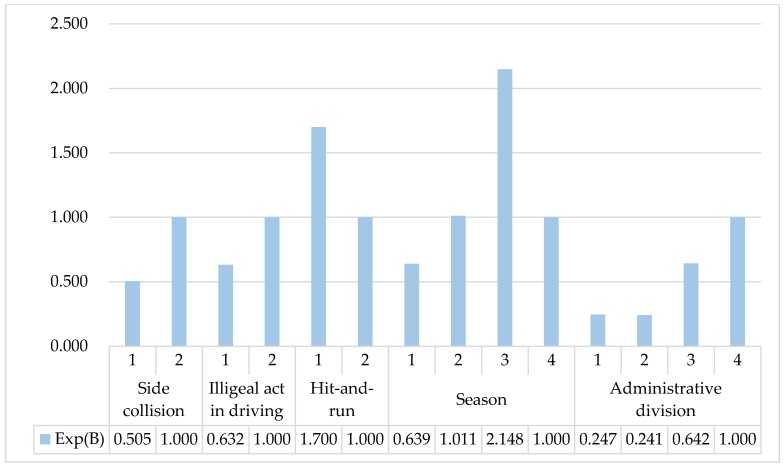
Exp(B) of different values of significant influencing factors on passenger vehicle accidents.

Among the 20 influencing factors analyzed in passenger vehicle accidents, five influencing factors show statistically significant correlations with the interpreted variable (*p* < 0.05), which are side collision, illegal act while driving, hit-and-run, season and administrative division. The results are shown in Figure 1 and analyzation is carried out one by one for the relevant influencing factors:

For the influencing factor of side collision, when its value is 1, its probability of causing death in traffic accidents is 0.505 times that when its value is 2. Therefore, when the collision type is non-side collision, it is more likely to lead to death in traffic accidents than side collision.

For the influencing factor of illegal act while driving, when its value is 1, its probability of causing death in traffic accidents is 0.632 times of that when its value is 2. Therefore, for illegal and irregular driving behavior, irregular driving behavior is more likely to lead to deaths in traffic accidents than illegal driving behavior.

For the influencing factor of hit-and-run, when its value is 1, its probability of causing death in traffic accidents is 1.700 times of that when its value is 2. Therefore, hit-and-run behavior is more likely to lead to death in traffic accidents than non-hit-and-run behavior.

For the influencing factor of season, the probability of causing death in traffic accidents of value 1, 2 and 3 are, separately, 0.639 times, 1.011 times, 2.148 times of that when its value is 4. Therefore, autumn is most likely to lead to death in traffic accidents among the four seasons. The second most deadly seasons in traffic accidents are summer and winter, with almost the same probability. Spring is the season with the lowest probability of causing death in traffic accidents among four seasons.

For the influencing factor of administrative division, the probability of causing death in traffic accidents of value 1, 2 and 3 are, separately, 0.247 times, 0.241 times, 0.642 times of that when its value is 4. Therefore, city highway is the area that is most likely to cause death in traffic accidents among four different administrative divisions. The probability of causing death in traffic accidents in outer suburbs is about 2.7 times that in suburban areas and main urban areas. Suburban and main urban areas own the lowest probability of causing death in traffic accidents among four different administrative divisions.

### 4.2. Freight Vehicle Accidents

**Figure 2:**
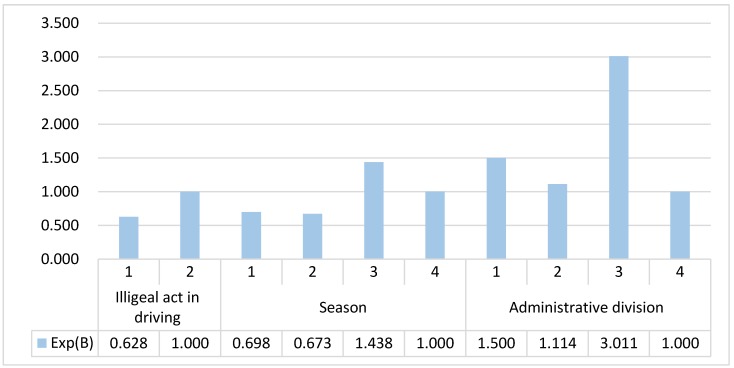
Exp(B) of different values of significant influencing factors on freight vehicle accidents.

Among the 20 influencing factors analyzed in freight vehicle accidents, three influencing factors show statistically significant correlation with the interpreted variable (*p* < 0.05), which are illegal act while driving, season and administrative division. The results are shown in Figure 2 and analyzation is carried out one by one for the relevant influencing factors:

For the influencing factor of illegal act while driving, when its value is 1, its probability of causing death in traffic accidents is 0.628 times of that when its value is 2. Therefore, for illegal and irregular driving behavior, irregular driving behavior is more likely to lead to death in traffic accidents than illegal driving behavior.

For the influencing factor of season, the probability of causing death in traffic accidents of value 1, 2 and 3 are, separately, 0.698 times, 0.673 times, 1.438 times of that when its value is 4. Therefore, autumn is most likely to lead to death in traffic accidents among the four seasons. The second most deadly season in traffic accidents is winter. Spring and summer are the seasons with the lowest probability of causing death in traffic accidents, owning almost the same possibility.

For the influencing factor of administrative division, the probability of causing death in traffic accidents of value 1, 2 and 3 are, separately, 1.500 times, 1.114 times, 3.011 times of that when its value is 4. Therefore, outer suburbs are most likely to cause death in traffic accidents among four different administrative divisions. The second most deadly administrative division in traffic accidents is main urban area. Suburban area ranks the third in probability of causing death in traffic accidents. City highway owns the lowest probability of causing death in traffic accidents among four different administrative divisions.

## 5. Discussion

### 5.1. Common Points of Passenger and Freight Vehicle Accidents

According to the calculation results above, illegal act while driving is the only common influencing factor for injury severity of passenger and freight vehicle accidents. When the value of illegal act is equal to 2, the probability of death accident occurrence in passenger vehicle accidents and freight vehicle accidents are, separately, 1.582 times and 1.592 times that when the illegal act value is equal to 1. Those numbers show that irregular acts while driving for both passenger and freight vehicles cause a higher possibility of death occurrence in traffic accidents than illegal act while driving. When a deeper analysis of irregular acts is undertaken, the types of irregular act can be divided into several types: irregular driving operation, no attention to pedestrians at no-signal intersections, driving with a slight overspeed, etc. In traffic accidents in Shenyang, irregular acts account for 75.4% and 63.8% for passenger and freight vehicle accidents separately. Weng and Meng [24] discussed risky driving behavior of vehicles at work zones and similar results were found in our study. Ma et al. [7] also found irregular driving behavior such as aggressive driving is related to traffic accident deaths. Therefore, it is necessary to legislate some irregular driving behaviors into illegal driving behaviors to make drivers realize that their irregular driving behaviors can be illegal and reduce irregular driving behavior consequences.

### 5.2. Differences of Passenger and Freight Vehicle Accidents

#### 5.2.1. Factors Only Influencing Injury Severity in Passenger Vehicle Accidents

Side collision is a significant influencing factor for the injury severity of passenger vehicle accidents, but not for the injury severity of freight vehicle accidents. In the case of passenger traffic accidents, considering that passenger vehicles are of full or main responsibility, the lethal probability of non-side collision accidents is 1.980 times of that of side collision accidents. Therefore, for passenger traffic accidents, more attention should be paid to non-side collision accidents. Non-side collision accidents of passenger vehicles usually include frontal collision, rear-end collision, frontal scraping, rear scraping, etc. To reduce the death rate of these kinds of accidents, it is necessary to add warning signs and other warning facilities to alert the drivers at the positions where there may be a frontal or rear-end collision with non-motor vehicles or pedestrians, such as the road sections where non-motor vehicles and motor vehicles are not clearly separated. For freight vehicles, because they are usually operated with heavy loads representing a large weight, when freight vehicles are mainly or fully responsible for traffic accidents, the collision energy produced by both side collision and non-side collision is much larger than that of passenger vehicles, so more attention should be paid to limiting the speed of freight vehicles to reduce their impact energy under various types of collisions.

Hit-and-run is a significantly influencing factor for the injury severity of passenger vehicle accidents, but not for the injury severity of freight vehicles. In the case of passenger traffic accidents, considering that passenger vehicles are fully or mainly responsible, the lethal probability of hit-and-run accidents is 1.700 times of that of non-hit-and-run accidents. Therefore, for passenger traffic accidents, more attention should be paid to hit-and-run accidents. To reduce the occurrence of hit and run accidents, it is necessary to reinforce driver education and punishment. In the aspect of driver education, the legal education for drivers should be strengthened, and the harmfulness of hit-and-run accidents should be emphasized in both driver training and examination and lifelong education for drivers; In the aspect of driver punishment, it is necessary to increase the penalties and economic punishment for the driver’s hit-and-run behavior, which will serve as a warning for hit-and-run behavior. Aidoo et al. [25] studied road and environmental characteristics on hit-and-run accidents. Roshandeh et al. [26] compared influencing factors in hit-and-run accidents of distracted and non-distracted drivers.

In summary, factors only influencing death in passenger vehicle accidents include illegal acts while driving and hit-and-run behavior. Both factors belong to the driver attributes. In comparison with freight vehicle drivers, the driving experience of passenger vehicle drivers are usually less. In China, drivers who can drive freight vehicles should obtain a corresponding passenger vehicle license first and then pass the freight vehicle tests [27]. Therefore, it is necessary to pay more attention to driver education and road infrastructure, improve the requirements of passenger drivers, and ensure the protection of vulnerable groups such as non-motor vehicles and pedestrians.

#### 5.2.2. Factors Presenting Different Results on Passenger and Freight Vehicle Accidents

Season is a significant influencing factor for the injury severity of both passenger and freight vehicle accidents. For passenger vehicle accidents, the probability of causing death in traffic accidents of spring, summer and autumn are 0.639 times, 1.011 times, 2.148 times that of winter, respectively; for freight vehicle accidents, the probability of causing death in traffic accidents of spring, summer and autumn 0.698 times, 0.673 times, 1.438 times that of winter, respectively. Therefore, autumn is the most likely season to result in death in traffic accidents among the four seasons for both passenger and freight vehicles. According to the historically statistical data of weather in Shenyang (http://lishi.tianqi.com/shenyang/index.html), the maximum and minimum temperatures in September and October of 2015, 2016 and 2017 in Shenyang are shown in Table 4:

According to the statistical data of Shenyang city, autumn has the biggest temperature drop in the four seasons. The drastic temperature change will make the tire pressure change. In addition, due to the sharp decline in night temperature to almost zero or lower than zero degrees Celsius, i.e., ice point, the road is typically wet or frozen in the early morning the next day, but the drivers are less prepared for the bad road driving environment than they are in winter, which increases the possibility of traffic death. The second most deadly season in traffic accidents is winter for both passenger and freight vehicles. The main reason is that the traffic environment of Shenyang in winter is bad in comparison to other seasons, and there is often unexpected rain and snow [28], which leads to the roads becoming wet and slippery, coupled with low visibility [29], hence increasing the road traffic death possibility. In summer, the probability of death in passenger vehicle accidents is almost the same as that in winter, and in freight vehicle accidents is 0.673 times as that in winter. One possible explanation is that for Shenyang, since the poor road environment in the winter was unsuitable for practice, many inexperienced passenger drivers choose to practice driving in the short summer season after a long winter period which may lead to more serious traffic accidents; whilst freight transportation is largely concerned with business transportation, and its injury severity is not strongly related to driver attributes. Spring is the season with the lowest death rate of traffic accidents for both passenger and freight traffic. The main reason is that spring is the season with the best driving environment among the four seasons.

Administrative division is a significant influencing factor for the injury severity of both passenger and freight vehicle accidents. Sun et al. [30]. also found administrative division is a significant influencing factor for injury severity. For passenger vehicle accidents, the probability of causing death in traffic accidents of main urban area, suburban area and outer suburbs are, 0.247 times, 0.241 times, 0.642 times of city highway, respectively; for freight vehicle accidents, the probability of causing death in traffic accidents of urban area, suburban area and outer suburbs are 1.500 times, 1.114 times, 3.011 times of city highway, respectively. For passenger traffic, city highway has the highest death rate of traffic accidents among the four regions. Therefore, it is necessary to strengthen the infrastructure of city highway for passenger vehicle drivers and actively improve the auxiliary driving technology, to reduce the number of serious accidents caused by the lack of driving experiences of passenger vehicle drivers. However, for freight traffic, city highway has the lowest death rate of traffic accidents among the four regions, which is mainly due to the control of freight vehicles by the Ministry of Transport of China, which adopts several control technologies including freight electronic weighing and electronic toll collection to monitor the traffic. Meanwhile, the access level of freight drivers is higher than that of passenger drivers. The area with the highest death rate in freight vehicle accidents is the outer suburbs, which is 2.007 times of main urban area and 2.703 times of suburban area. In passenger vehicle accidents, the death rate of outer suburbs is the second highest, which is 2.526 times of main urban area and 2.589 times of suburban area. For freight and passenger traffic, outer suburbs are areas with high death rate of traffic accidents. One possible explanation is that the economic conditions in the outer suburbs are poor, the road conditions are poor, and the lack of electronic monitoring make the death rate higher than main urban area and suburban area.

## 6. Conclusions

Based on the data from the city of Shenyang, 1647 traffic accidents from 2015 to 2017 in which motor vehicles are of full or main responsibility are analyzed, including 1164 traffic accidents caused by passenger vehicles and 483 traffic accidents caused by freight vehicles. Twenty influencing factors from the aspects of accident, driver, time, space and environmental attributes are conducted to find their statistical connection with injury severity by using a binary logistic regression model.

According to the calculation results, illegal acts while driving are the only common influencing factor for injury severity of passenger and freight vehicle accidents. When the value of illegal acts is equal to 2, the probability of death accident occurrence in passenger vehicle accidents and freight vehicle accidents are 1.582 times and 1.592 times that of value 1, respectively. Factors including side collision, hit-and-run, season and administrative division show differences in their influence on passenger and freight vehicle accidents. The factors of side collision and hit-and-run are significant influencing factors for the injury severity of passenger vehicle accidents, but not of freight vehicle accidents. Season and administrative division present different results on influencing passenger and freight vehicle accidents. Measures to address these problems, including driver education, road infrastructure improvement, etc., should be implemented to reduce the injury severity of passenger and freight vehicle accidents

## Figures and Tables

**Table 1:** Definition of different values of injury severity and influencing factors.

Interpreted Variable (Y)	Definition
Injury severity (Y)	0 = No death; 1 = Death accident
**Explanatory variables (X)**	
**Accident attributes**	
Type of motor vehicle (X_1_)	1 = Small-sized passenger or freight vehicles;
	2 = Medium-sized passenger or freight vehicles;
	3 = Large-sized passenger or freight vehicles
Frontal collision (X_2_)	1 = Yes; 2 = No
Side collision (X_3_)	1 = Yes; 2 = No
Rear-end collision (X_4_)	1 = Yes; 2 = No
Accident liability (X_5_)	1 = Full responsibility; 2 = Main responsibility
**Driver attributes**	
Gender (X_6_)	1 = Man driver; 2 = Woman driver
Age (X_7_)	Continuous variable showing the age
Driving years (X_8_)	Continuous variable showing driving years
Illegal act in driving (X_9_)	1 = Yes; 2 = No, irregular act only
Owning a qualified driver’s license (X_10_)	1 = Yes; 2 = No
Hit-and-run * (X_11_)	1 = Yes; 2 = No
**Time attributes**	
Weekday or weekend (X_12_)	1 = Weekday; 2 = Weekend
Season (X_13_)	1 = Spring (April, May);
	2 = Summer (June, July, August);
	3 = Autumn (September, October);
	4 = Winter (November, December, January, February, March)
Time Interval (X_14_)	1 = 0:00–6:00; 2 = 6:00–12:00; 3 = 12:00–18:00; 4 = 18:00–24:00
Rush hour (X_15_)	1 = Yes (7:00–9:00, 17:00–19:00); 2 = No (Other time periods)
**Spatial attributes**	
Administrative division (X_16_)	1 = Main urban area; 2 = Suburban area; 3 = Outer suburbs; 4 = City highway
Location of road cross section (X_17_)	1= Motor vehicle lane; 2 = Mixed lane of motor vehicles and non-motor vehicles; 3= Non-motor vehicle lane; 4 = Others
Physical isolation of road (X_18_)	1 = Isolation between motor and non-motor vehicle; 2 = No isolation; 3= Central isolation; 4 = Central isolation and isolation between motor and non-motor vehicle
Pavement condition (X_19_)	1 = Good; 2 = Others
**Environmental attributes**	
Weather (X_20_)	1 = Sunny; 2 = Others

*: ”Hit-and-run” refers to the behavior of the full or main responsible party escaping from the scene after a road traffic accident.

**Table 2:** Percentage of different values of variables in passenger and freight vehicle accidents.

Interpreted Variable (Y)	Value	Percentage of Passenger Vehicle Accidents (%)	Percentage of Freight Vehicle Accidents (%)
Injury severity (Y)	0	62.5	41.6
	1	37.5	58.4
**Explanatory variables (X)**			
**Accident attributes**			
Type of motor vehicle (X_1_)	1	91.4	34.6
	2	1.1	3.7
	3	7.5	61.7
Frontal collision (X_2_)	1	6.8	7.2
	2	93.2	92.8
Side collision (X_3_)	1	36.7	38.3
	2	63.3	61.7
Rear-end collision (X_4_)	1	9.5	17.0
	2	90.5	83.0
Accident liability (X_5_)	1	64.1	58.6
	2	35.9	41.4
**Driver attributes**			
Gender (X_6_)	1	91.1	99.4
	2	8.9	0.6
Age (X_7_)		Continuous variable	
Driving years (X_8_)		Continuous variable	
Illegal act in driving (X_9_)	1	24.6	36.2
	2	75.4	63.8
Owning a qualified driver’s license (X_10_)	1	94.9	98.1
	2	5.1	1.9
Hit-and-run (X_11_)	1	13.0	6.2
	2	87.0	93.8
**Time attributes**			
Weekday or weekend (X_12_)	1	71.1	74.9
	2	28.9	25.1
Season (X_13_)	1	10.2	21.3
	2	25.9	30.0
	3	29.9	17.4
	4	33.9	31.4
Time Interval (X_14_)	1	10.2	18.0
	2	25.9	25.5
	3	29.9	34.2
	4	33.9	22.4
Rush hour (X_15_)	1	24.8	19.5
	2	75.2	80.5
**Spatial attributes**			
Administrative division (X_16_)	1	30.8	18.2
	2	44.8	44.3
	3	21.4	30.8
	4	3.1	6.6
Location of road cross section (X_17_)	1	68.0	69.2
	2	4.8	16.8
	3	16.2	6.0
	4	10.9	8.1
Physical isolation of road (X_18_)	1	1.6	2.3
	2	78.8	75.8
	3	17.2	20.3
	4	2.4	1.7
Pavement condition (X_19_)	1	99.5	98.8
	2	0.5	1.2
**Environmental attributes**			
Weather (X_20_)	1	90.0	88.8
	2	10.0	11.2

**Table 3:** Results of binary logistic regression analysis.

Variables	Passenger Vehicle Accidents		Freight Vehicle Accidents	
	*p*	Exp(B)	*p*	Exp(B)
**Side collision**	0.000		-	
1		0.505		
2		#		
**Illegal act in driving**	0.004		0.025	
1		0.632		0.628
2		#		#
**Hit-and-run**	0.005		-	
1		1.700		
2		#		
**Season**	0.000		0.047	
1		0.639		0.698
2		1.011		0.673
3		2.148		1.438
4		#		#
**Administrative division**	0.000		0.000	
1		0.247		1.500
2		0.241		1.114
3		0.642		3.011
4		#		#

“-” indicates that the variable is not significant. “#” indicates the reference value of Exp(B).

**Table 4:** Temperature of Shenyang from 2015 to 2017.

Year	Month	Maximum Temperature (°C)	Minimum Temperature (°C)
2015	September	30	7
	October	26	−5
2016	September	27	3
	October	26	−9
2017	September	28	3
	October	23	−5
